# The antioxidant role of pumpkin (*Cucurbita pepo*) seed extract against acute reproductive toxicity by uranyl acetate in male rats

**DOI:** 10.5455/javar.2023.j720

**Published:** 2023-12-31

**Authors:** Ghusoon Abdul Kareem Neamah, Muna Abdul Sahib Alkhfaji, Heba Saleh Shaheed

**Affiliations:** Department of Pathology and Poultry Diseases, College of Veterinary Medicine, Al-Qassim Green University, Babylon, Iraq

**Keywords:** Antioxidant, histopathology, pumpkin (*Cucurbita pepo*), rat

## Abstract

**Objective:**

The main goal of the study was to find whether pumpkin (*Cucurbita pepo*) extract has any preventive or antioxidant properties against acute uranyl acetate (UA)-induced reproductive cytotoxicity.

**Material and Methods:**

Four groups each, including 10 adult male rats, were randomly assigned. (GI): the control group was given 1 ml of purified water orally for 30 days. (GII): Rats were given orally, a single dose of 150 mg/kg b.w. UA (GIII): Rats consumed 40 mg/kg b.w. of pumpkin seed extract (PSE) orally every day for 30 days. (GIV): Rats received a single dose (150 mg/kg b.w.) of UA plus a daily oral dose of PSE (40 mg/kg b.w.) for 30 days. Animal sacrifice was used for oxidative stress and histopathological study.

**Results:**

Showed significantly (*p* ≥ 0.001) elevated malondialdehyde levels in the GII group (6.19 ± 0.4), while GIII and GIV showed no significant differences. Glutathione peroxidase showed a significant (*p* ≥ 0.001) decrease (2.55 ± 0.2) in the GII group, while in groups (GIII and GIV), it showed a significant (*p* ≥ 0.001) increase (4.61 ± 0.16, 4.28 ± 0.032), respectively. The histopathological study for GII groups showed sloughing of epithelial cells lining the seminiferous tubules with a decrease in the number of spermatozoa in some tubules. Many sections revealed hyperplasia of the epithelial cells lining the seminiferous tubules with necrosis. The GIII and GIV groups showed normal histological structures with an increase in spermatogenesis in the testes and epididymis tissues.

**Conclusion:**

We concluded that UA causes oxidative stress and histopathological alterations in the rat reproductive system. Pumpkin extract plays a role in improving the activity of the reproductive system.

## Introduction

Uranium is a silvery-white heavy metal having chemical and radioactive characteristics that make it beneficial to business, the military, and industry but poisonous at high enough concentrations. Since the formation of our planet, uranium has been a component of its crust. It is found in various concentrations in the rock, soil, air, and water of the area, and it enters the body through the air, food, and water [1]. All three of the uranium isotopes that make up natural uranium—99.284% U-238, 0.711% U-235, and 0.005% U-234—are radioactive. Depleted uranium has a prolonged physical half-life; therefore, it can linger in the environment for a very long time in places including the soil, groundwater, plants, and animals [2].

Some studies on rodents have shown a detrimental effect of uranium on reproductive processes. More precisely, earlier findings have shown that, with prolonged exposure, seminiferous tubules‘ histological consequences are less severe, including interstitial and reduction in sperm count per testis and male fertility [3]. On the other hand, findings of testicular histological abnormalities with seminiferous tubule deformations have been linked to acute exposure [4]. A significant leaf and seed vegetable with a stellar reputation in traditional medicine is the pumpkin (*Cucurbita pepo*) [5]. Photochemical sterols, antioxidant vitamins such as carotenoids, proteins, phytosterols, polyunsaturated fatty acids (PUFAs), tocopherol, and trace minerals such as zinc and selenium are all abundantly found in the seeds [6]. In addition, it contains phenolic substances such as tyrosol, vanillin, ferulic acid, vanillic acid, and luteolin [7].

In addition, it has been demonstrated to have significant amounts of tocopherol, which interprets its antioxidant activity and subsequently suggests that it would be able to lessen lipid peroxidation [8]. Pumpkin seed extract (PSE) has therapeutic benefits for a variety of illnesses, including immune regulation, reproductive health, and therapeutic advantage [9]. In addition to helping to avoid arteriosclerosis, high blood pressure, and heart disease, PSEs antioxidant capabilities may also increase fertility [10,11].

## Material and Methods

### Ethical approval

All procedures were performed in accordance with the laws governing animal care and the guidelines on animal welfare of the pathology department, College of Veterinary Medicine, Al-Qasim Green University, Babylon, Iraq. The ethical criteria were followed in the conduct of this investigation established by the local Animal Welfare Committee**.**

### Experimental animals

Adult male rats, weighing between 180 and 230 gm and aged 8–10 weeks, were purchased from the animal house of the Al-Qasim Green University/College of Veterinary Medicine. During the experiment, the ideal housing circumstances for the animals were a humidity level of 55%–65%, a temperature range of 20°C–25°C, and a 12-h light/dark cycle. The animals were given a pellet meal and unlimited access to water. Each group of rats was kept in a plastic cage with bedding made of hardwood chips. Every 2 days, the bedding was changed to maintain a clean environment.

### Preparation of C. pepo L. seed extract

The extraction of the pumpkin seeds was carried out according to the maceration technique described by Siddig [12]. 50 gm of powdered seeds were put in a conical flask. 500 ml of 70% ethanol was poured on. A magnetic stirrer was used to periodically agitate the liquid for 24 h before filtering it through filter paper No. 1 from Whatman. The filter media was heated to room temperature and evaporated. Until usage, the finished extract was kept at 4°C.

### Dose preparation for C. pepo L. seed extract

40 mg/kg body weight of PSE was taken orally every day and dissolved in 1 ml of distilled water [13].

### Chemicals

Uranyl acetate (UA) ([UO_2_ (CH_3_CO_2_) _2_H_2_O)] H_2_O (yellow to green crystals) was obtained from SPI-Chem (USA).

### Dose calculation for UA

The dose was calculated depending on the value of LD_50_ 150 mg/kg bw. Administered to the rats by oral intubations after being dissolved in normal saline using a stomach tube as a single dose [14].

### Oxidative stress study

#### Blood collection

For all groups of rats, blood samples were drawn straight from the heart using a disposable syringe. They were then placed in test tubes without any anticoagulant, left to coagulate, and utilized for biochemical testing. To extract the serum from the coagulated blood samples, centrifugation was done for 5 min at 3,000 rpm. The serum was then frozen at −20°C until it was employed in the biochemical assay malondialdehyde (MDA), glutathione (GSH).

#### Serum lipid peroxidation measurement (MDA)

We measured serum lipid peroxidation. According to Buege and Aust [15].

#### Measuring serum-reduced GSH

It was made in accordance with the Greenwald [16].

#### Histopathological study

Testes and epididymis were removed from the animals after scarification with chloroform and fixed right away in a 10% buffered formalin solution before being histologically examined and integrated with paraffin. The slides were then routinely hematoxylin and eosin were used to stain the slides after the histological sections were cut using a rotary microtome [17].

### Statistical analysis

SAS was used to examine the data (Statistical Analysis System, version 9.1). *p* 0.001 was taken to be statistically significant. Statistically significant and least significant differences were performed to evaluate significant differences among means [18].

## Results

### Oxidative stress study

The results of the oxidative stress study are seen in Table 1. The MDA level was significantly (*p* ≥ 0.001) increased in the group treated with UA (GII) (6.19 ± 0.4) compared with the control group (GI) (1.25 ± 0.04). While in GIII and GIV (treated with 40 mg/kg b.w. PSE and single dose (150 mg/kg b.w.) UA, plus oral dosage each day of PSE (40 mg/kg b.w. for 30 days), respectively, there were no significant differences in Constance with the control group (1.18 ± 0.04, 1.16 ± 0.03). However, the result of glutathione peroxidase was a significant (*p* ≥ 0.001) decrease (2.55 ± 0.2) in the group treated with UA (GII) compared with the control group (4.02 ± 0.087). While groups (GIII and GIV) were treated with 40 mg/kg b.w. PSE and single dose (150 mg/kg b.w.) UA, plus oral dosage each day of PSE (40 mg/kg b.w. for 30 days), respectively, showed significant (*p* ≥ 0.001) increased (4.61 ± 0.16, 4.28 ± 0.032), respectively, compared with the control group (4.02 ± 0.087) and GII (2.55 ± 0.2).

**Table 1.** The (Mean ± SE) levels of MDA and (GSH) glutathione peroxidase (µmol/l) in the serum of rat for different experiment groups.

Groups	MDA (Mean ± SE)	GSH (Mean ± SE)
GI	1.25 ± 0.04^b^	4.02 ± 0.087^b^
GII	6.19 ± 0.4^a^	2.55 ± 0.2^c^
GIII	1.18 ± 0.04^b^	4.61 ± 0.16^a^
GIV	1.16 ± 0.03^b^	4.28 ± 0.032^ab^
*p*-value	129.68	38.27
Pr > F	≥ 0.001	≥ 0.001

Different small litters mean significant differences at level (*p* ≥ 0.001) between the experimental groups.

### Histopathological study

#### Testes

**Figure 1.**
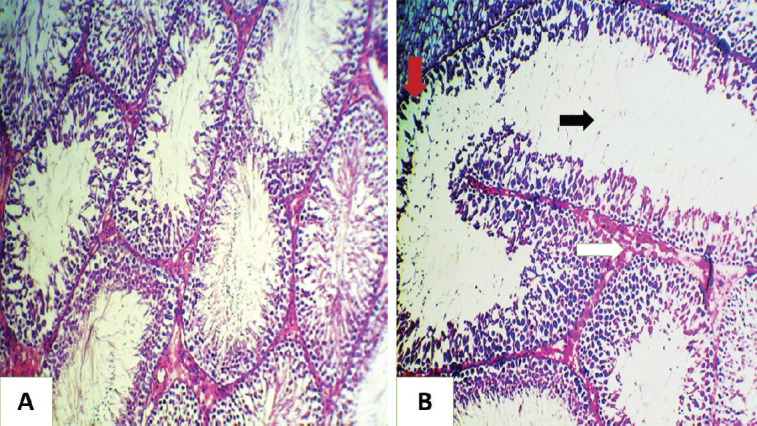
Histopathological section of rat testes (A) for the control group shows normal histological structures. (B) for GII group showed sloughing of epithelial cells lining the seminiferous tubules (red arrow) with a decrease in number of spermatozoa in some tubules (black arrow), also mild interstitial thickening (white arrow) ( H&E stain, 200×).

**Figure 2.**
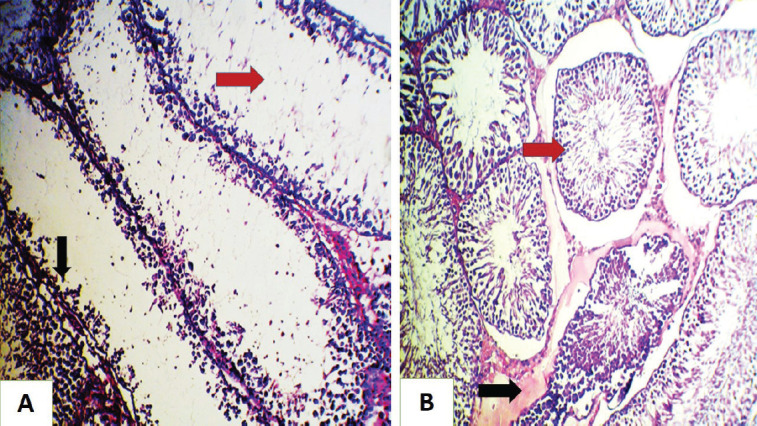
Histopathological section of rat testes (A) for the GII group shows necrosis of epithelial cells lining the seminiferous tubules (loss of spermatogonia) with inflammatory cells infiltration, (red arrow) decrease in number of spermatozoa in some tubules (black arrow). (B) for the GII group shown collapsed of the seminiferous tubule wall (papered with the irregular wall) (red arrow), with interstitial inflammatory exudate (black arrow) (H&E stain, 200×).

**Figure 3.**
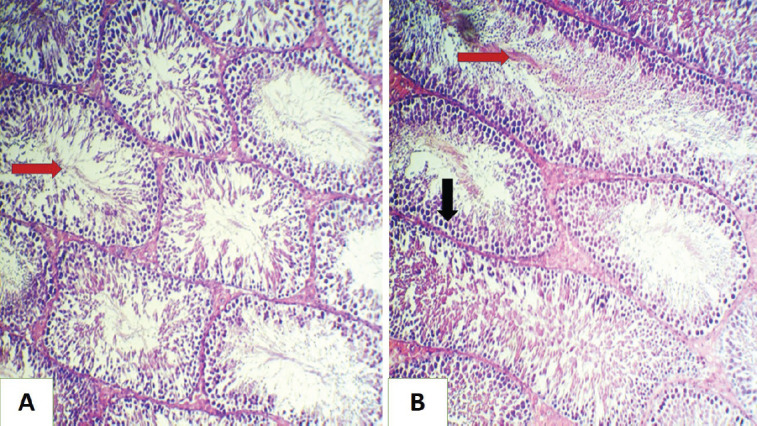
Histopathological section of rat testes (A) for the GIII group shows normal histological structures with an increase in spermatogenesis (red arrow). (B) for GIV group showed increased production of spermatozoa (red arrow), with regeneration of the tubular epithelia (black arrow) (H&E stain, 200×).

**Figure 4.**
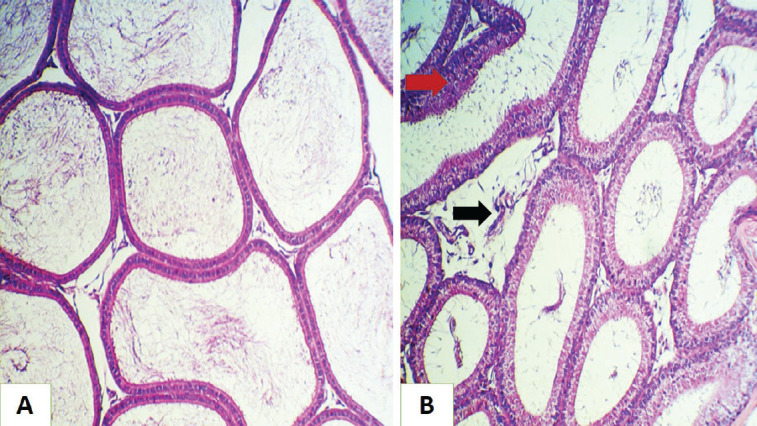
Histopathological section of rat epididymis (A) for the GI group showed normal histological structures. (B) for the GII group showed hyperplasia of epithelial cells lining the seminiferous tubules (red arrow ), with the presence of sloughing epithelia inside the lumen (black arrow) (H&E stain, 200×).

The histopathological changes for GI groups showed normal histological structure (Figs. 1A and 4A). The main pathological lesions in the rat test section for GII groups were the sloughing of epithelial cells lining the seminiferous tubules with a decrease in the number of spermatozoa in some tubules, as well as mild interstitial thickening (Fig. 1B) and the seminiferous tubules‘ epithelial cells were necrotizing with inflammatory cell infiltration, with a decrease in the number of spermatozoa in some tubules (Fig. 2A). Also collapsed was the seminiferous tubule wall (papered with an irregular wall), with interstitial inflammatory exudate (Fig. 2B). However, the most examined histological sections for GIII groups showed normal histological structures with an increase in spermatogenesis (Fig. 3A). In addition, GIV groups showed increased production of spermatozoa and regeneration of the tubular epithelia (Fig. 3B).

#### Epididymis

**Figure 5.**
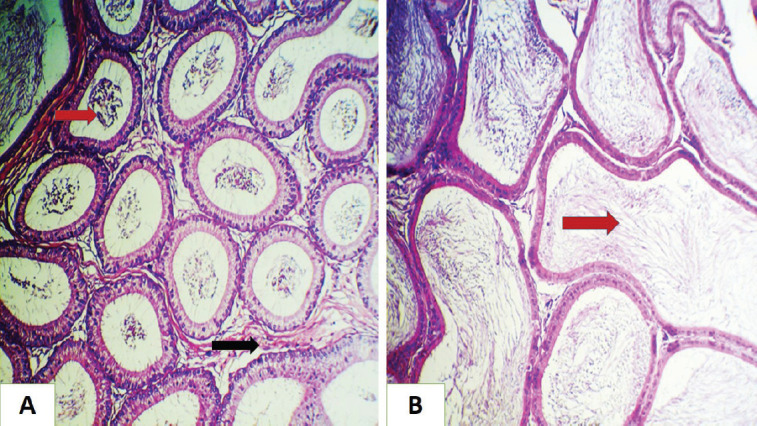
Histopathological section of rat epididymis (A) for GII group shows inflammatory cells inside the seminiferous tubules (red arrow) (appear empty from the sperm) with mild interstitial edema (black arrow). (B) for the GIII group showed normal tubular epithelia with increased numbers of spermatozoa (red arrow) (H&E stain, 200×).

**Figure 6.**
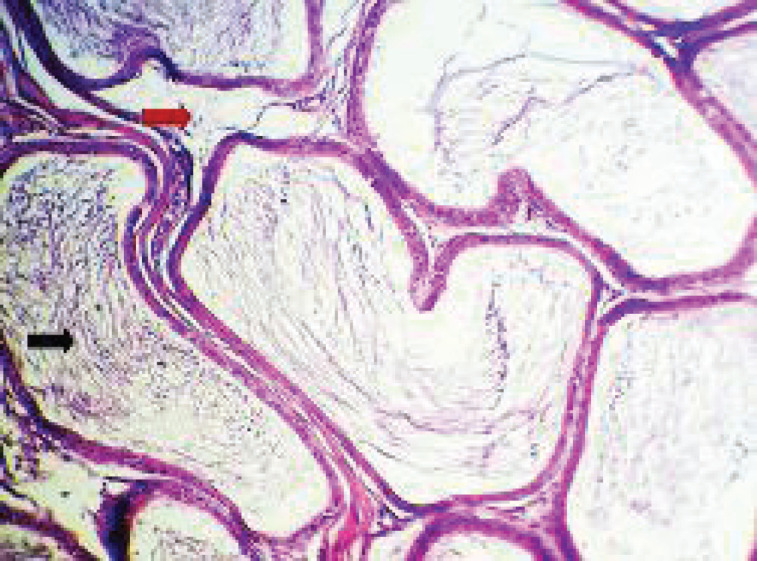
Histopathological section of rat epididymis for GIV group shows mild interstitial edema (red arrow), with normal tubular epithelia and increased numbers of spermatozoa (black arrow) (H&E stain, 200×).

The results for the section of rat epididymis for the GII group show hyperplasia of epithelial cells lining the seminiferous tubules with the presence of sloughing epithelia inside the lumen (Fig. 4B) and inflammatory cells inside the seminiferous tubules (which appear empty from the sperm) with mild interstitial edema (Fig. 5B). In the GIII group, the most examined section showed normal tubular epithelia with increased numbers of spermatozoa (Fig. 5B). While in GIV, the histological section showed mild interstitial edema with normal tubular epithelia and increased numbers of spermatozoa (Fig. 6).

## Discussion

### Oxidative stress study

PUFAs undergo lipid peroxidation, which produces MDA. The concentration of MDA in tissues can be used to evaluate the level of lipid peroxidation [19]. MDA is created when reactive oxygen species (ROS) break down polyunsaturated lipids. This result may be explained by uranium‘s capacity to inhibit the activity of antioxidant enzymes such as superoxide dismutase (SOD), catalase, and GSH, which may ultimately result in the induction of oxidative stress in tissue. The current investigation found that serum MDA levels had significantly increased and there was a decrease in GSH levels in the GII groups. The buildup of free radicals under these circumstances would lead to oxidative damage to biomolecules such as lipids, which would then result in lipid peroxidation. This finding is consistent with research showing that uranium can cause oxidative stress and lipid peroxidation. However, UA can break down electron transport chains, which causes a reduction in glutathione and lipid peroxidation. The production of hydroxyl radicals from superoxide anions and hydrogen peroxide can be sped up by the use of uranium as a catalyst for the Fenton reaction. Lipid peroxidation, which raises the concentration of PUFAs, is thought to be caused by hydroxyl radicals. The testes are vulnerable to damage from ROS, and the level of ROS may also serve as an indirect indicator of the toxicity of uranium [20,21].

GSH levels dramatically increased, whereas MDA levels significantly dropped in GIII and GIV. This result may be explained by the pumpkin seed oil extract antioxidant compounds; phenolic acids, polyphenols, and flavonoids are examples of compounds that scavenge free radicals such as peroxide and hydro-peroxide of lipid hydroxyl and so prevent the oxidative mechanisms that lead to degenerative illnesses [8]. Vitamin E and polyphenolics are abundant in pumpkin seed oil (PSO). Phenolic chemicals have anti-inflammatory, anti-mutagenic, anticarcinogenic, and antioxidant effects [22]. Similar to this, polyphenolics lessen cell oxidative injury brought on by free radicals and guard against DNA damage caused by oxidants. This outcome was in line with several studies [23–25].

### Histopathological study

The main pathological lesions in rat testes and epididymis sections for GII groups were sloughing of epithelial cells lining the seminiferous tubules with a decrease in the number of spermatozoa in some tubules, mild interstitial thickening, and epithelium lining cell necrosis in the seminiferous tubules (loss of spermatogonia), with inflammatory cell infiltration and a decrease in the number of spermatozoa in some tubules. Also collapsed was the seminiferous tubule wall (papered with an irregular wall), with interstitial inflammatory exudate. In the epididymis, the section showed hyperplasia of epithelial cells lining the seminiferous tubules with the presence of sloughing epithelia inside the lumen and inflammatory cells inside the seminiferous tubules (appear empty from the sperm) with mild interstitial edema. This outcome may be related to the ability of uranium to cause reproductive harm due to its chemical and radioactive properties [20]. In earlier research, it was also demonstrated that uranium builds up in the reproductive organs. Acute inhalation exposure to high levels of uranium dust (instantaneous concentration up to 1,800 mg/m^3^) may be harmful to rat testicles by having fewer sperm and a higher rate of spermatogenesis cells dying off [26]. However, the histopathological results in the GIII and GIV groups showed normal histological structures with an increase in spermatogenesis; in the epididymis, the exanimated section showed normal tubular epithelia with an increase in spermatozoa. In addition, GIV groups showed increased production of spermatozoa with regeneration of the tubular epithelia, and epididymis showed mild interstitial edema with normal tubular epithelia and increased numbers of spermatozoa. This is a result of PSEs protective and improving properties, which may be related to its significant antioxidant and free radical-scavenging capacity [27], and PSE high in carbs may have boosted sperm survival and motility by boosting glucose metabolism, which leads to the synthesis of pyruvate and Adenosine triphosphate (ATP) [28]. This improved sperm survival and motility was likely due to the stimulating effect of ATP synthesis. Pyruvate is the preferred substrate known to be essential for sperm cell activity and survival. Since only sperm cells with increasing motility engage in the process of fertilization, sperm cell movement is essential for fertilization [29].

In addition, arginine, an amino acid that is found in large quantities in pumpkin seeds, is metabolized from the sperm-motility-related compounds putrescine, spermidine, and spermine [30]. PSO was discovered to be a rich source of antioxidants and healthy nutrients such as carotenes, lutein, gamma and P-tocopherols, phytosterols, chlorophyll, selenium, and zinc in studies [31] and [32]. PUFAs and essential fatty acids are two additional nutrients present in PSO. Linoleic acid, which is present in PSO, is known to enhance membrane fluidity, encourage osmosis, and support intracellular and extracellular gas exchange [33]. In most cases, the generated ROS and antioxidant scavengers are in equilibrium. Vitamins C, E, and A are among the antioxidants abundant in PSE [34]. Magnesium, phosphorus, manganese, copper, and iron, which are essential for the male reproductive system, are also abundant in pumpkin seeds.

Vitamin A prevents lipid peroxidation in the testis, which encourages spermatogenesis and enhances the morphological maturation of the epididymis‘s epithelial cells, according to Fukuchi et al. [35]. This antioxidant property may have prevented lipid peroxidation from harming the spermatozoa, which is a strong likelihood. In addition, the monounsaturated fatty acid oleic acid decreases the testes and epididymis‘ sensitivity to lipid peroxidation [36].

PSO leaves, according to Sheweita et al. [30], have healing actions on the testes and enhance sperm production. In a study by Akang et al. [37], it was found that testosterone levels and semen quality were both increased in male rats using PSO, which also had a preventative impact on alcohol-induced testicular injury. Our histopathology findings were consistent with earlier research [38,39]. It is also possible that PSE is to blame for accelerating the transition from an inflammatory reaction to an anti-inflammatory response. According to Mokhtar et al. [40], PSO exhibited an inhibitory effect on tumor necrosis factor (TNF). These modifications affected the ability of phytochemicals to reduce inflammation [41]. Saponins reduce the activity of the cyclooxygenase-2 (COX-2) enzyme and reduce the generation of TNF, according to Shao et al. [42].

## Conclusion

We concluded that UA has the potential to cause oxidative stress and histopathological changes in the male rat reproductive system. PSE plays a significant role in improving the activity of the reproductive system and acts as an antioxidant against the oxidative stress brought on by the toxicity of uranium acetate.

## List of Abbreviations

ATP, Adenosine triphosphate; COX-2, cyclooxygenase-2; GSH, Glutathione; MDA, Malondialdehyde; PSE, Pumpkin seed extract; PSO, Pumpkin seed oil; PUFAs, Polyunsaturated fatty acids; ROS, Reactive oxygen species; SOD, Superoxide dismutase; UA, Uranyle acetate.
